# Supplementary material for: Does Hessian Data Improve the Performance of Machine Learning Potentials?
Source: J Chem Theory Comput. 2025 Jul 2;21(14):6698–710. doi: 10.1021/acs.jctc.5c00402 (PMC12288065; doi:10.1021/acs.jctc.5c00402)
Supplement: Supplementary file 1 [file ct5c00402_si_001.pdf]

Supporting Information: Does Hessian data improve the performance of machine learning potentials?

Austin Rodriguez,[†] Justin S. Smith,[‡] and Jose L. Mendoza-Cortes[*,†,¶]

†*Department of Chemical Engineering & Materials Science, Michigan State University, East Lansing, Michigan 48824, United States.*

‡*NVIDIA Corp., San Tomas Expy, Santa Clara, California 95051, United States.*

¶*Department of Physics and Astronomy, Michigan State University, East Lansing, MI, 48823, USA.*

E-mail: jmendoza@msu.edu

A Datasets and code

OpenReACT-CHON-EFH — **O**pen **Re**action Dataset of **A**tomic **C**onfigura**T**ions comprising **C**,**H**,**O**,**N** with **E**nergies, **F**orces, and **H**essians

A.1 Where to find the Datasets

The dataset **OpenReACT-CHON-EFH** used in this study is available on Figshare at:

`https://doi.org/10.6084/m9.figshare.29189858`

A.2 Where to find the Code

The codebase developed and used in this work is available on GitHub under the `MLFFwithHessians` repository:

`https://github.com/mendozacortesgroup/MLFFwithHessians`

B Training Dataset Description

In Figure S1, we provide a comprehensive overview of the dataset. These figures show the distribution and diversity of chemical species within the dataset, shedding light on the types of reaction and chemical environments captured. Specifically, we include histograms that illustrate the distribution of different elements and display the number of occurrences for each type of atom.

In addition, we present histograms that categorize the number of single, double, triple, and aromatic bonds in the reactants and products in Figure S2. These histograms provide insight into the bond types prevalent in the reactions, highlighting the nature of the chemical transformations captured in the dataset. By presenting these figures, we aim to provide a comprehensive overview of the dataset and its suitability in training ML/AI models and improving the accuracy of the Hessian-Trained ML-FF.

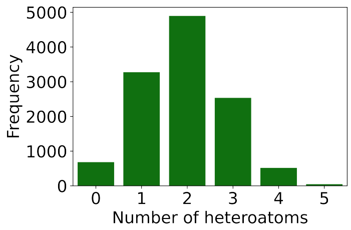

(a) Heteroatom population

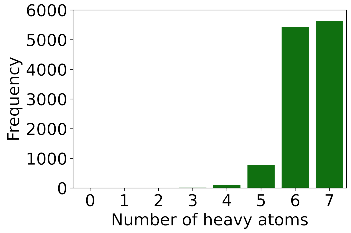

(b) Heavy atom population

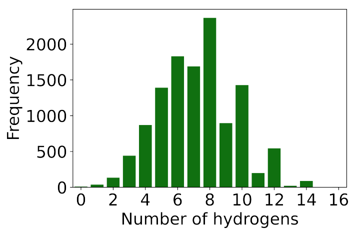

(c) Hydrogen population

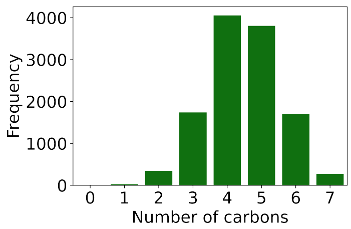

(d) Carbon population

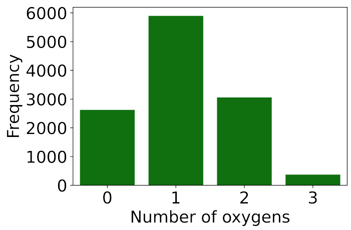

(e) Oxygen population

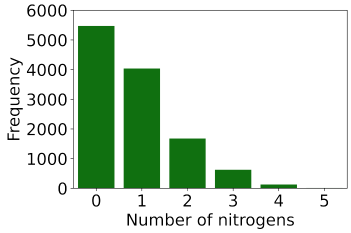

(f) Nitrogen population

Figure S1: Overview of the composition of molecules in the dataset we will use to train the ML models. Histograms of heteroatoms, heavy atoms, and each atom type in molecules involved in the reactions of the dataset. Heteroatoms are considered to be any atom except carbon (C) or hydrogen (H).

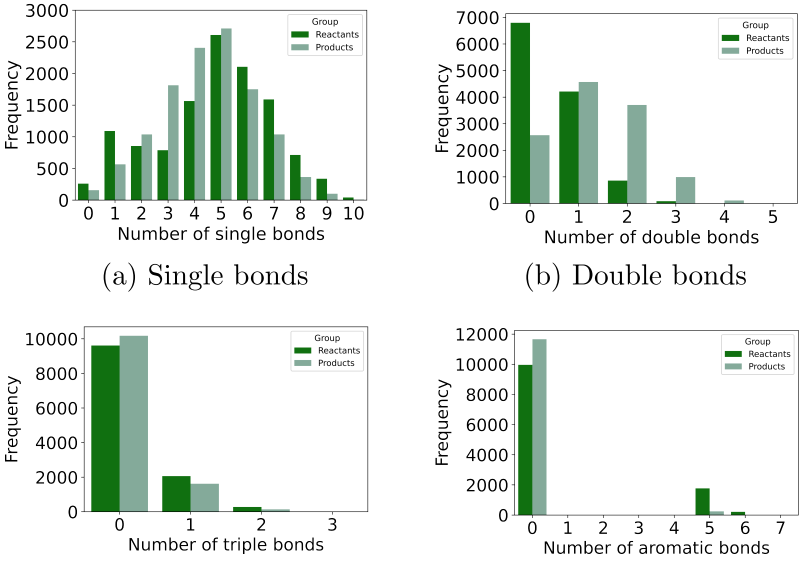

(a) Single bonds (b) Double bonds

(c) Triple bonds (d) Aromatic bonds

Figure S2: Histograms containing the frequency of molecules with each number of single, double, triple, or aromatic bonds.

For training, we have calculated the Hessian at each point, which is defined as:

$$H_E = \begin{bmatrix} \frac{\partial^2 E}{\partial x_1^2} & \frac{\partial^2 E}{\partial x_1 \partial y_1} & \frac{\partial^2 E}{\partial x_1 \partial z_1} & \frac{\partial^2 E}{\partial x_1 \partial x_2} & \cdots & \frac{\partial^2 E}{\partial x_1 \partial z_n} \\ \frac{\partial^2 E}{\partial y_1 \partial x_1} & \frac{\partial^2 E}{\partial y_1^2} & \frac{\partial^2 E}{\partial y_1 \partial z_1} & \frac{\partial^2 E}{\partial y_1 \partial x_2} & \cdots & \frac{\partial^2 E}{\partial y_1 \partial z_n} \\ \frac{\partial^2 E}{\partial z_1 \partial x_1} & \frac{\partial^2 E}{\partial z_1 \partial y_1} & \frac{\partial^2 E}{\partial z_1^2} & \frac{\partial^2 E}{\partial z_1 \partial x_2} & \cdots & \frac{\partial^2 E}{\partial z_1 \partial z_n} \\ \frac{\partial^2 E}{\partial x_2 \partial x_1} & \frac{\partial^2 E}{\partial x_2 \partial y_1} & \frac{\partial^2 E}{\partial x_2 \partial z_1} & \frac{\partial^2 E}{\partial x_2^2} & \cdots & \frac{\partial^2 E}{\partial x_2 \partial z_n} \\ \vdots & \vdots & \vdots & \vdots & \ddots & \vdots \\ \frac{\partial^2 E}{\partial z_n \partial x_1} & \frac{\partial^2 E}{\partial z_n \partial y_1} & \frac{\partial^2 E}{\partial z_n \partial z_1} & \frac{\partial^2 E}{\partial z_n \partial x_2} & \cdots & \frac{\partial^2 E}{\partial z_n^2} \end{bmatrix} \tag{S1}$$

where H_E is called the Hessian matrix, E represents the total energy of the system, and x_i, y_i, and z_i represent each of the Cartesian coordinates of the atomic position of the atom i.

C MLIP Architecture

The ANI-1 model uses modified Behler and Parrinello symmetry functions to capture the intricate chemical environments that surround individual atoms.[1,2] These vectors then become input for a specialized form of High Dimensional Neural Network Potentials (HD-NNP).[3] Different NNPs are deployed for each atom type, each equipped with its own set of weights and biases. Architecturally, these HD-NNPs are structured as feed-forward neural networks, featuring multiple hidden layers and a variety of neurons. The outputs of each of these NNPs correspond to a partition per atom of the molecular potential energy. These values are summed up to obtain the potential energy.

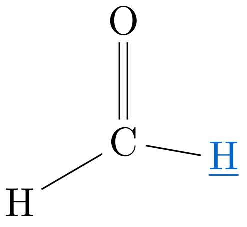

(a) Formaldehyde with C_s symmetry. The two nonequivalent hydrogens are displayed in different colors.

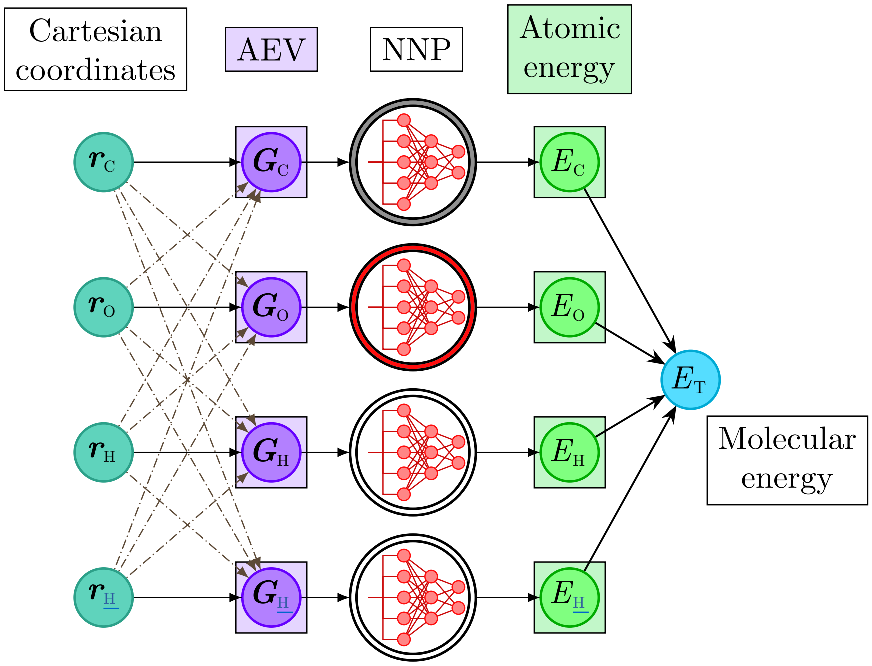

(b) A diagram of an HD-NNP for the formaldehyde molecule. The G-blocks calculate an *atomic environment vector*, AEV, which is fed to the NNP. The molecular energy is the sum of the individual atomic energies. This figure was adapted from figure 2 of Behler's paper.[2]

Figure S3: Use of an array of NNPs to calculate the energy of a formaldehyde molecule. The formaldehyde molecule has three atom types, and therefore, three distinct NNPs are necessary. Each NNP is indicated by its ring color, and has its own set of G functions and its own architecture.

D Single Transition State Training

The xyz-formatted molecular geometry data for the reactant, transition state, and product of the reaction used in the single-TS training are presented below.

```
13
Reactant
C                 1.76908200    0.61766900    0.45171600
O                 0.36988700    0.50871900    0.42121600
C                -0.08120500   -0.61521500   -0.30205800
C                -1.59249100   -0.50273600   -0.37993000
O                -1.97798800    0.68798800   -1.02483500
H                 2.01367900    1.51467300    1.02468900
H                 2.23015800   -0.25486600    0.93926600
H                 2.18921100    0.71183100   -0.56146800
H                 0.34588800   -0.61102800   -1.31773600
H                 0.22082200   -1.55054000    0.19726500
H                -1.99865900   -1.33489900   -0.96248500
H                -2.00892300   -0.56304100    0.63778800
H                -1.47946100    1.39144400   -0.58730300

13
Transition State
C                 1.10145000    0.62209100    0.49341400
O                 0.87410500   -0.65128500    1.07463600
C                 0.15253600   -1.30831400    0.09222800
C                -0.98348800   -0.38881300   -0.25144600
O                -0.68621400    1.24171600   -1.45195100
H                 1.31717700    1.34194100    1.28796500
H                 1.93978200    0.57254500   -0.22443100
H                 0.03395600    1.21570600   -0.68633100
H                 0.76052500   -1.52975300   -0.80418800
H                -0.23399100   -2.27098400    0.47284000
H                -1.69037300   -0.72898700   -1.00454300
H                -1.43064800    0.09057200    0.61359400
H                -1.34670400    1.90114800   -1.18191400

13
Product
C                 0.72266900    0.22324600    0.12956500
O                 1.36449700   -0.83581000    0.85470300
C                 0.29209400   -1.74302100    0.55981100
C                -0.49894200   -0.65196000   -0.18137300
O                -0.90831800    2.26604900   -1.48616600
```

H	0.54062100	1.09954700	0.76515900
H	1.30410900	0.53415100	-0.74610800
H	-0.13413500	2.82747300	-1.36715600
H	0.62824700	-2.58323500	-0.06077500
H	-0.17038200	-2.13841400	1.47241700
H	-0.69097700	-0.82569300	-1.24036400
H	-1.42578300	-0.34401100	0.30719000
H	-1.39232000	2.35211100	-0.65743700

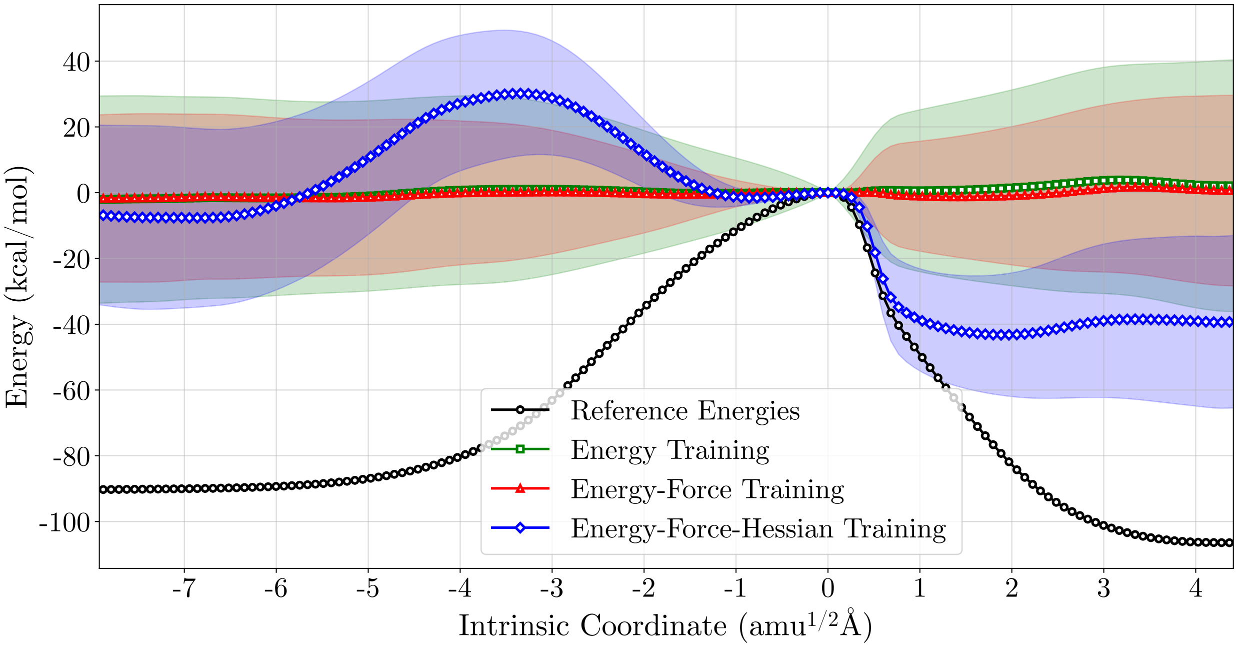

Figure S4: Energy predictions of molecular structures in IRC path of a reaction by models trained on only the TS point of the same reaction. The black circles correspond to the energies obtained by DFT calculations, which are considered to be the ground truth for our models. The colored markers represent the average energy predictions of the models trained to fit to energies; energies and forces; and energies, forces, and Hessian matrix of the data. The filled areas represent the standard deviation for each prediction by the ensemble of models.

E Ensemble Predictions

In this section, we present the ensemble predictions of our models for the energy values along the Intrinsic Reaction Coordinate (IRC) of a single reaction. Ensembles of models are used to enhance the robustness and reliability of the predictions by averaging the noise and reducing the variance inherent in single-model predictions.

For each type of model—energy fitting, energy-force fitting, and energy-force-Hessian fitting, we generate predictions using an ensemble of 100 models. Each model within the ensemble is represented by a line in the plots, with different fitting types distinguished by specific colors. This visualization allows for a clear comparison of the prediction performance and variability among the different fitting approaches.

Figure S5 below illustrates the ensemble predictions of the energy values along the IRC for the chosen reaction. By examining these plots, we can assess how consistently each type of model predicts energy values and identify any trends or discrepancies that may arise.

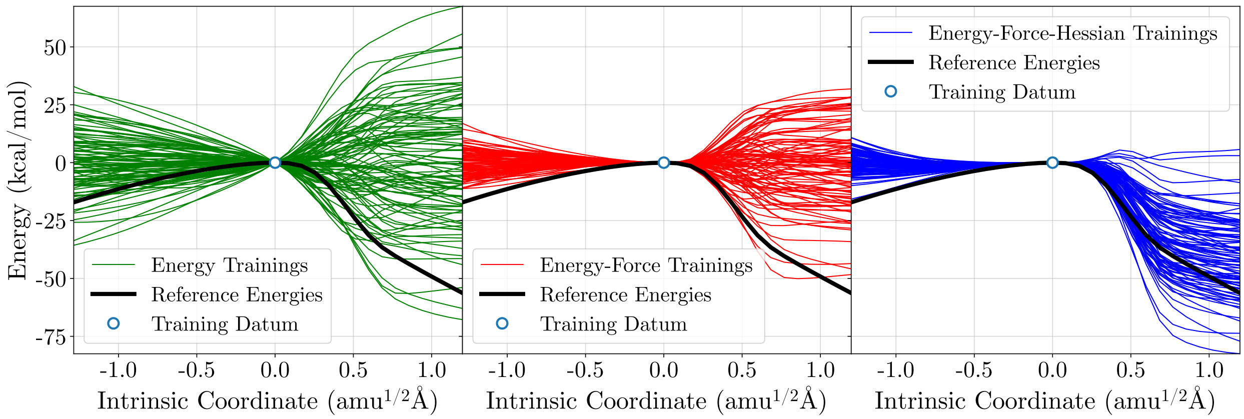

Figure S5: Ensemble predictions of energy values along the Intrinsic Reaction Coordinate (IRC) for a single reaction. The plot displays the predictions of three types of models: Energy Fitting (green lines), Energy-Force Fitting (red lines), and Energy-Force-Hessian Fitting (blue lines) compared to the reference energies (black line) and the Transition State (light blue circle). Each line represents the prediction of an individual model within an ensemble of 100 models for each fitting type.

F Correlation plots

Correlation plots serve as a valuable tool to assess the accuracy of our predictive models. They visually compare the reference values obtained from Density Functional Theory (DFT) calculations (x-axis) with the predicted values generated by our models (y-axis). In these graphs, the diagonal red dotted line represents the ideal case where the predicted values perfectly match the reference values (i.e. $y = x$). The closer the points are to this red dotted line, the more accurate the model predictions are. The density gradient in these plots indicates the concentration of data points, providing insight into areas where predictions are more consistent or where deviations are more frequent.

We have developed three types of models to enhance the prediction accuracy for different properties: Energy Fitting Models, Energy-Force Fitting Models, and Energy-Force-Hessian Fitting Models. All of these models were trained on the same dataset, which is composed of 35,087 reactants, transition states, and products that are part of 11,961 distinct reactions (R-TS-P dataset).

Each model is evaluated on three distinct datasets:

1. **R-TS-P Dataset**: This dataset includes reactants, transition states, and products for 11,961 reactions (35,087 individual structures).

2. **IRC Dataset**: This dataset consists of Intrinsic Reaction Coordinates (IRCs) for 2,000 reactions (225,963 individual structures).

3. **NMS Dataset**: This dataset contains perturbed structures obtained from Normal Mode Sampling (NMS) of the IRC structures (58,219 individual structures).

For each dataset and fitting type, the models produce three types of correlation plots, reflecting the predictions for energy, force, and Hessian values. These plots provide a comprehensive overview of the performance of the model in different datasets and prediction types.

F.1 Energy Fitting Models

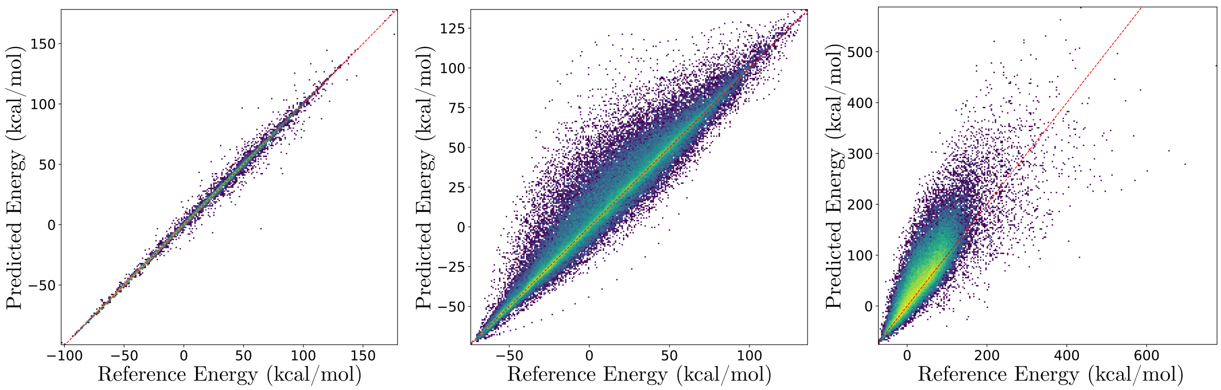

(a) R-TS-P Dataset - Energy (b) IRC Dataset - Energy (c) NMS Dataset - Energy

Figure S6: Energy Predictions of Energy Fitting Models

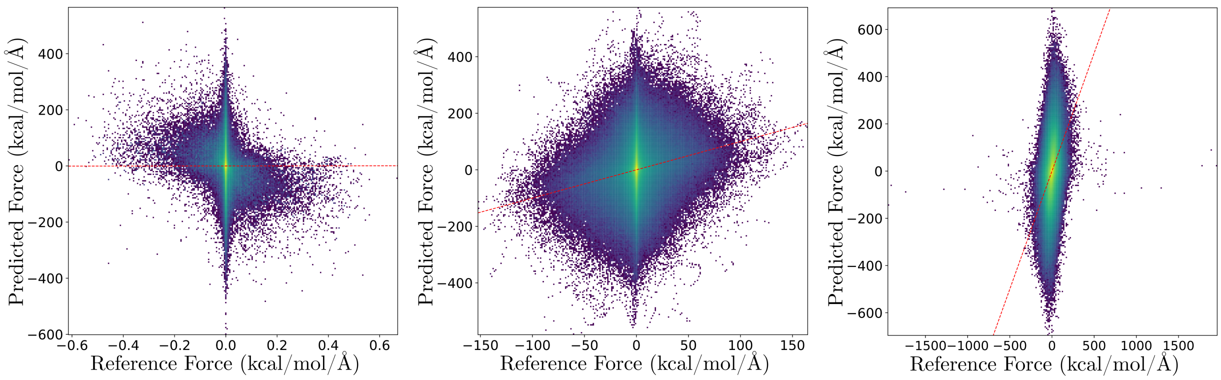

(a) R-TS-P Dataset - Force (b) IRC Dataset - Force (c) NMS Dataset - Force

Figure S7: Force Predictions of Energy Fitting Models

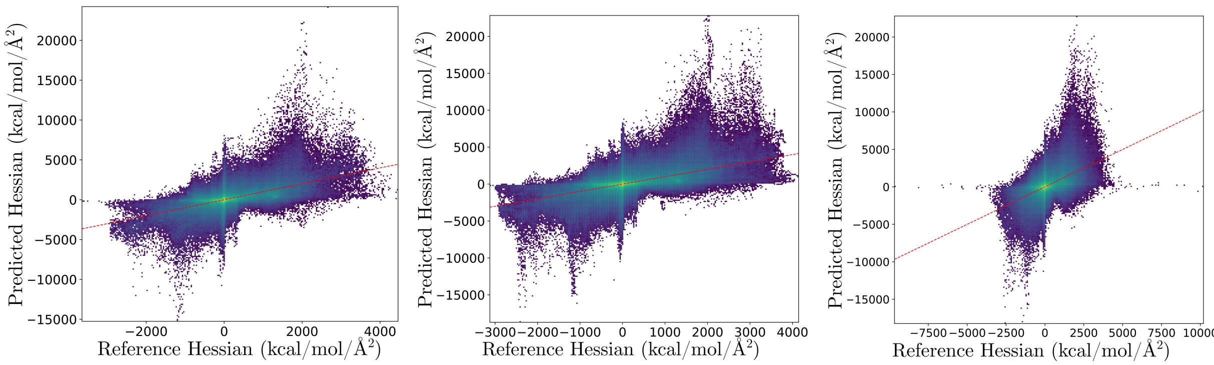

(a) R-TS-P Dataset - Hessian (b) IRC Dataset - Hessian (c) NMS Dataset - Hessian

Figure S8: Hessian Predictions of Energy Fitting Models

F.2 Energy-Force Fitting Models

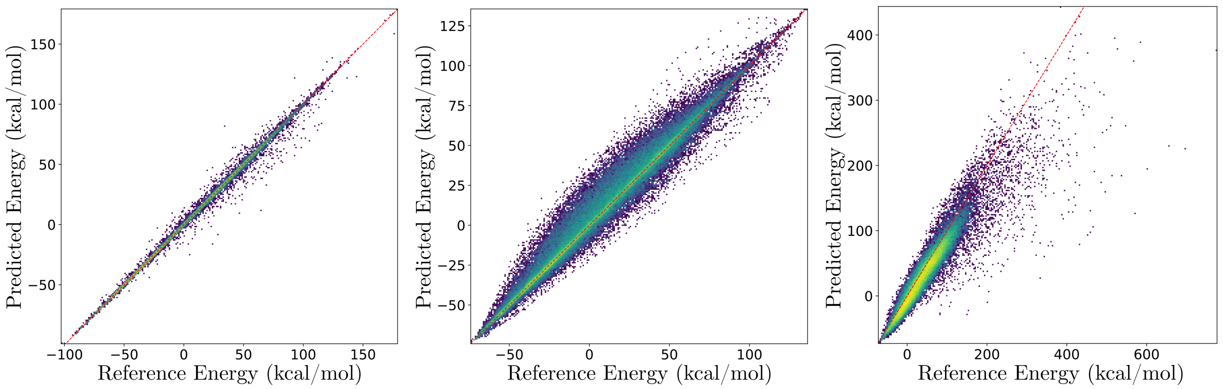

(a) R-TS-P Dataset - Energy (b) IRC Dataset - Energy (c) NMS Dataset - Energy

Figure S9: Energy Predictions of Energy-Force Fitting Models

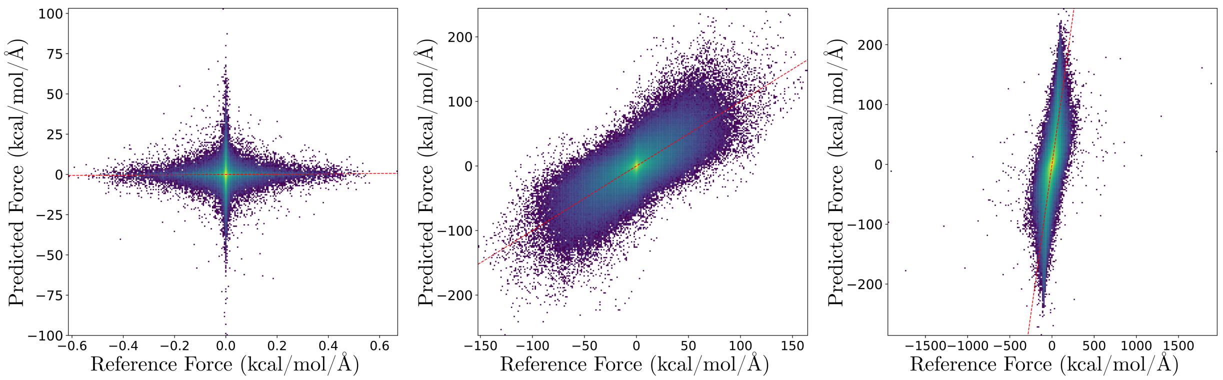

(a) R-TS-P Dataset - Force (b) IRC Dataset - Force (c) NMS Dataset - Force

Figure S10: Force Predictions of Energy-Force Fitting Models

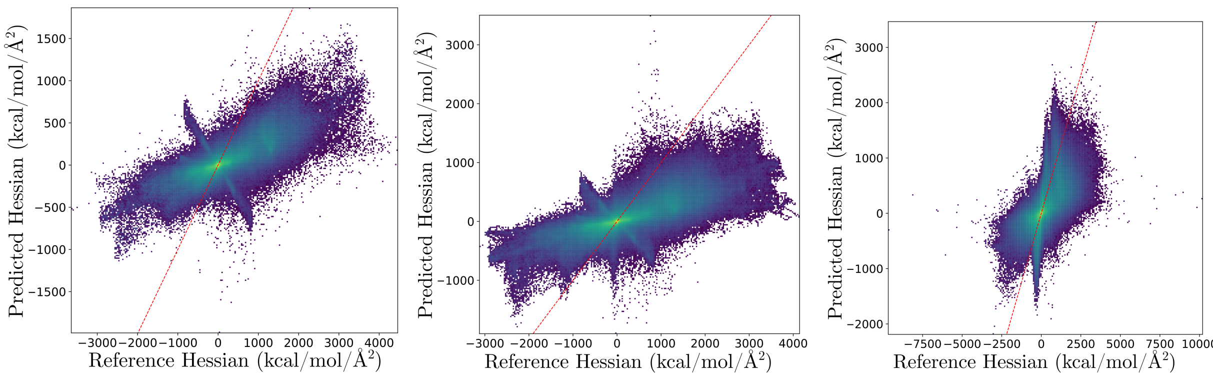

(a) R-TS-P Dataset - Hessian (b) IRC Dataset - Hessian (c) NMS Dataset - Hessian

Figure S11: Hessian Predictions of Energy-Force Fitting Models

F.3 Energy-Force-Hessian Fitting Models

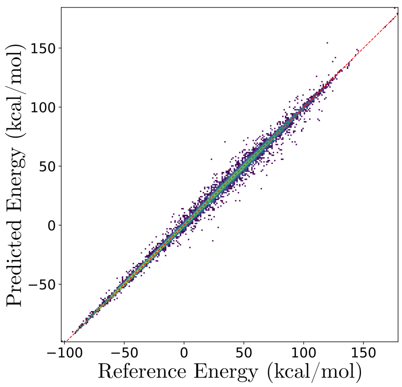

(a) R-TS-P Dataset - Energy

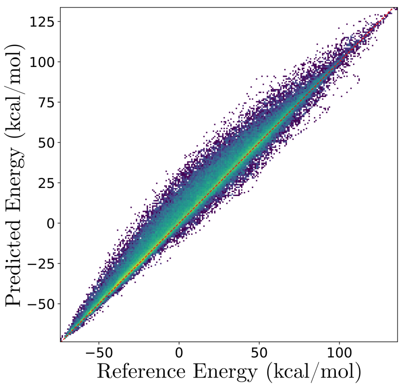

(b) IRC Dataset - Energy

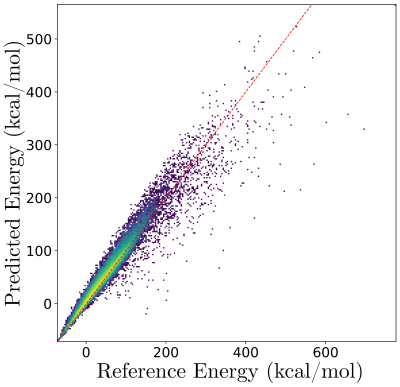

(c) NMS Dataset - Energy

Figure S12: Energy Predictions of Energy-Force-Hessian Fitting Models

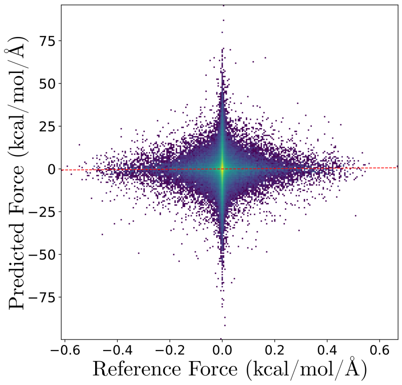

(a) R-TS-P Dataset - Force

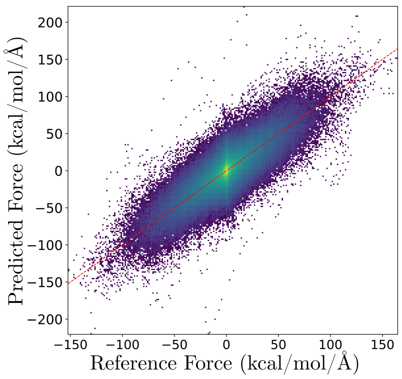

(b) IRC Dataset - Force

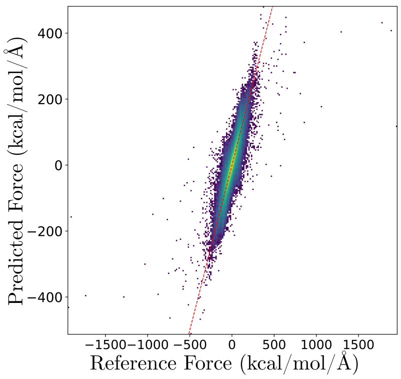

(c) NMS Dataset - Force

Figure S13: Force Predictions of Energy-Force-Hessian Fitting Models

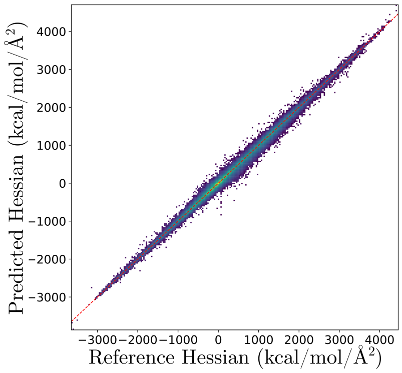

(a) R-TS-P Dataset - Hessian

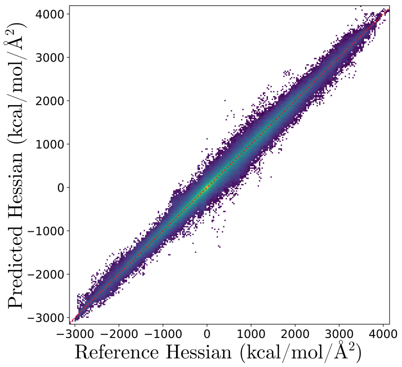

(b) IRC Dataset - Hessian

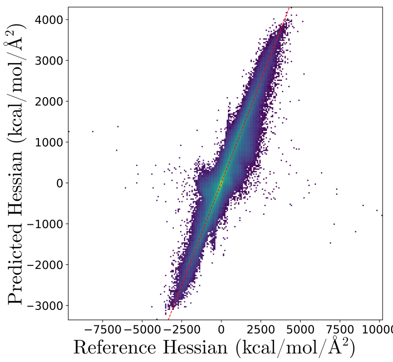

(c) NMS Dataset - Hessian

Figure S14: Hessian Predictions of Energy-Force-Hessian Fitting Models

G Stability in Molecular Dynamics Simulations

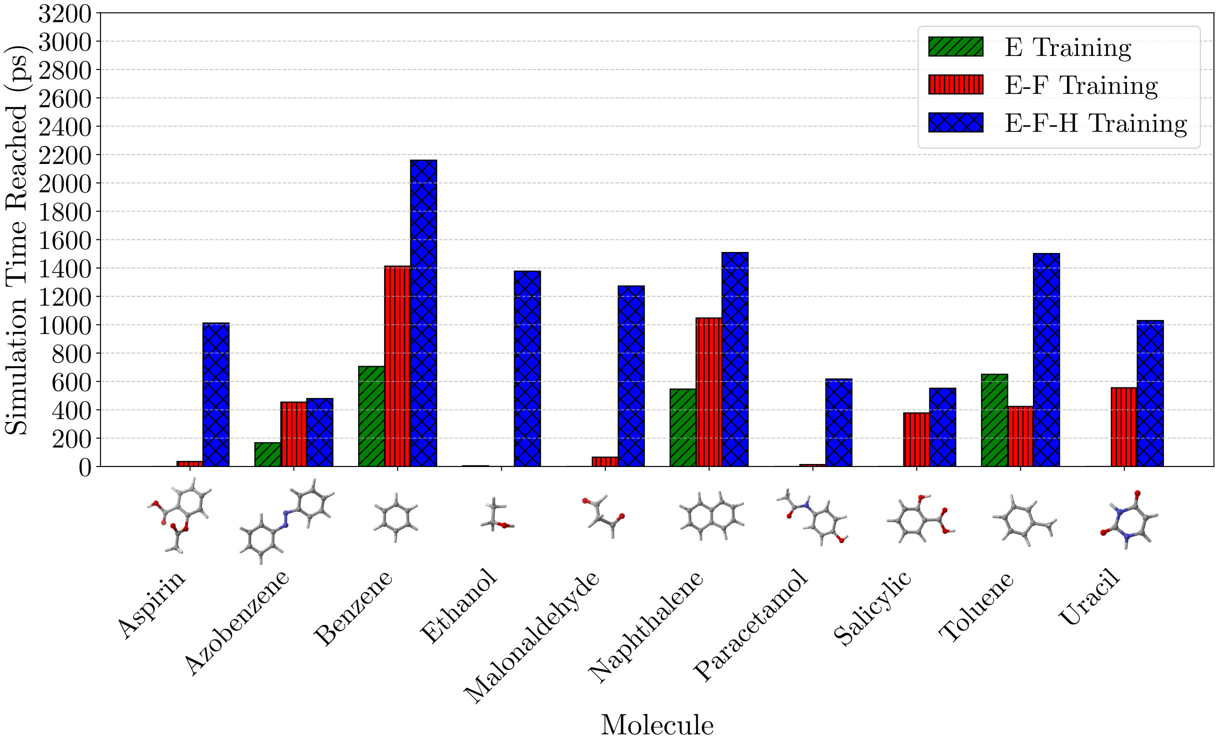

Figure S15: Simulation times reached before failure for each molecule in the MD17 dataset using ensembles of MLIP models trained with energy-only (E), energy-force (E-F), and energy-force-Hessian (E-F-H) loss functions.

The results, visualized in Figure S15, show the simulation times reached before failure for each molecule and highlight the significant differences between the three types of models. The E-F-H models exhibit substantially longer simulation times across all molecules, reflecting the enhanced stability achieved by incorporating Hessian information into the training process. The maximum temperatures and simulation times for each model type and molecule are summarized in Table SS1, providing a detailed quantitative comparison of the model performance.

Table S1: Maximum temperatures and total simulation times reached before failure for ensembles of MLIP models trained with energy-only (E), energy-force (E-F), and energy-force-Hessian (E-F-H) loss functions on each molecule in the MD17 dataset. Simulations that failed on optimization of the initial molecular geometry have a simulation time of 0.0 fs.

Molecule	Model	Temperature at Failure (K)	Simulation Time at Failure (fs)
Aspirin	E Training	0	0.0
	E-F Training	40	35157.0
	E-F-H Training	1015	1011677.0
Azobenzene	E Training	170	167332.5
	E-F Training	455	453821.0
	E-F-H Training	480	478923.0
Benzene	E Training	710	705498.5
	E-F Training	1415	1412553.5
	E-F-H Training	2160	2159901.0
Ethanol	E Training	5	4736.5
	E-F Training	0	0.0
	E-F-H Training	1380	1377289.0
Malonaldehyde	E Training	0	0.0
	E-F Training	70	65582.0
	E-F-H Training	1275	1272913.0
Naphtalene	E Training	550	545645.0
	E-F Training	1050	1047211.5
	E-F-H Training	1510	1508725.0
Paracetamol	E Training	0	0.0
	E-F Training	15	13492.5
	E-F-H Training	620	617084.5
Salicylic	E Training	0	0.0
	E-F Training	380	377569.5
	E-F-H Training	555	551635.5
Toluene	E Training	655	650086.0
	E-F Training	425	423360.5
	E-F-H Training	1505	1501559.0
Uracil	E Training	0	0.0
	E-F Training	560	555103.0
	E-F-H Training	1030	1029069.0

H NEB Analysis: Multi-Process Reaction

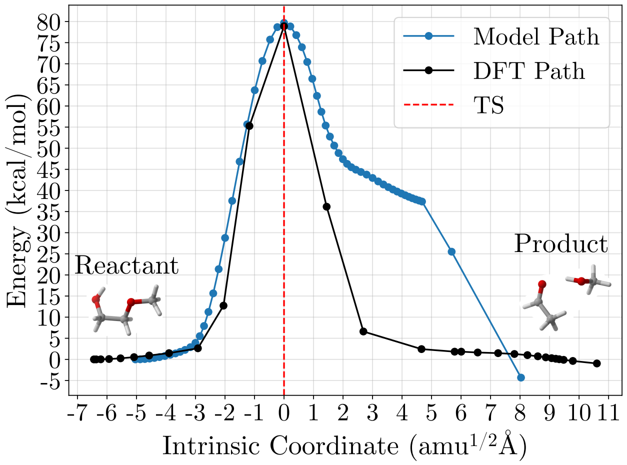

Figure S16: Energy profiles obtained from NEB calculations using models trained with an E-F-H loss functions (blue dots) alongside DFT reference values (black dots). The x axis represents the geometric distances between intermediates as Intrinsic Coordinates, where a reference value of zero was assigned to the TS geometry (dashed vertical red line). The y axis represents the energies in $\mathrm{kcal\,mol^{-1}}$. The E-only trained model and the E-F trained model were not able to converge in the NEB analysis of this reaction. This NEB represents a multi-process reaction where the EFH model does not completely reproduce. However, the energy barrier prediction compared to the DFT barrier is accurate. The atom coloring follows the CPK convention (red for oxygen, grey for carbon, and white for hydrogen).

To further assess the predictive accuracy and extrapolation capabilities of the Hessian-trained MLIP, we analyzed a multi-process reaction using Nudged Elastic Band (NEB) calculations. This reaction consists of multiple simultaneous processes, making it an ideal test case for evaluating how well the model captures reaction barriers and the overall shape of the potential energy surface (PES) beyond equilibrium structures.

While the activation energy predictions from the E-F-H model align well with DFT (78.88 $\mathrm{kcal\,mol^{-1}}$ from the model compared to 79.68 $\mathrm{kcal\,mol^{-1}}$ from DFT), significant deviations in the PES shape appear on the product side of the reaction coordinate. The MLIP-predicted energy profile does not smoothly reproduce the post-transition-state energy relaxation observed in DFT. Instead, the product basin is distorted, suggesting that the model does not fully capture the long-range relaxation effects or secondary reaction events that occur after the transition state.

I Data Efficiency for IRC Dataset

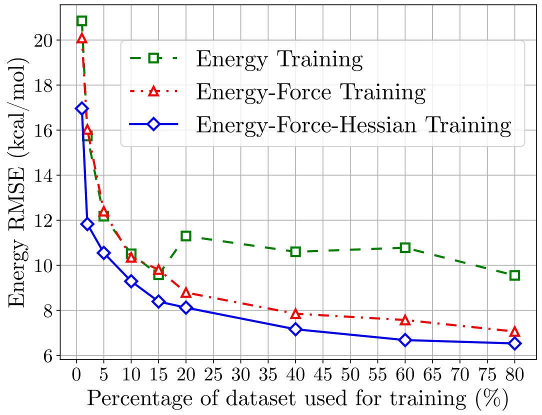

(a) Energy RMSEs

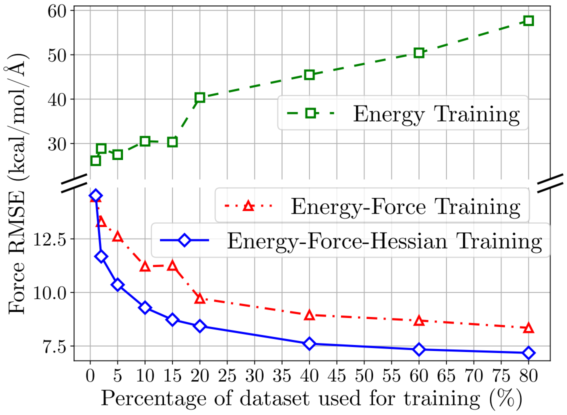

(b) Force RMSEs

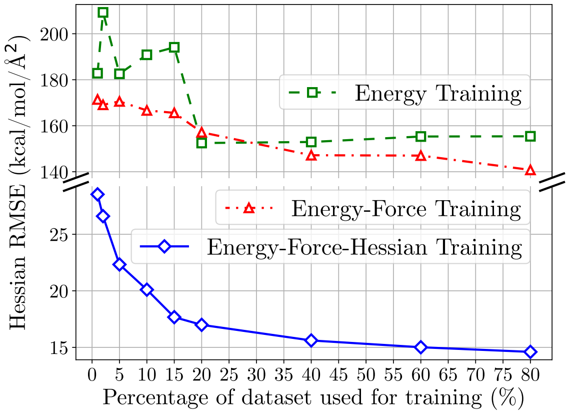

(c) Hessian RMSEs

Figure S17: Energy, force, and Hessian root mean squared errors and average training time per epoch versus training data volume. The models shown in these figures were trained on reactants, transition states, and product of 11,961 elementary chemical reactions and tested on IRC structures of the first 2,000 reactions in the dataset.

Examining the dashed blue lines in Figure S17a, it becomes evident that the inclusion of Hessian information significantly improves the data efficiency of the MLIP models. Remarkably, the model trained on energies, forces and Hessian matrices (energy-force-Hessian fitting model) attains a comparable RMSE in energy predictions of intermediate structures in IRC paths using just 20% of the total dataset volume, a performance parity achieved by the energy-force fitting model only when utilizing twice that amount of data. Furthermore, when the data volume for the Hessian energy force fitting model is increased to 40% of

the total volume of the data set, it almost achieves the RMSE performance in the energy predictions of the energy force fitting model trained with 80% of the dataset. These observations underscore a profound implication: incorporating Hessian data into the training process potentially doubles the data efficiency of MLIP models.

References

(1) Behler, J.; Parrinello, M. Generalized neural-network representation of high-dimensional potential-energy surfaces. *Physical Review Letters* **2007**, *98*, 1–4.

(2) Behler, J. Atom-centered symmetry functions for constructing high-dimensional neural network potentials. *Journal of Chemical Physics* **2011**, *134*.

(3) Behler, J. Constructing high-dimensional neural network potentials: A tutorial review. *International Journal of Quantum Chemistry* **2015**, *115*, 1032–1050.
